# Frailty and body composition predict adverse outcomes after emergency general surgery admission: a multicentre observational cohort study

**DOI:** 10.1308/rcsann.2024.0091

**Published:** 2024-11-21

**Authors:** P May-Miller, MAP Ligthart, AR Darbyshire, S Rahman, PH Pucher, NJ Curtis, MA West, for the Wessex Research Collaborative

**Affiliations:** ^1^ Salisbury NHS Foundation Trust, UK; ^2^ NUTRIM School of Nutrition and Translational Research in Metabolism, Maastricht University, The Netherlands; ^3^ Maastricht University Medical Centre, The Netherlands; ^4^ Dorset County Hospital NHS Foundation Trust, UK; ^5^ University Hospital Southampton NHS Foundation Trust, UK; ^6^ Portsmouth Hospitals University NHS Trust, UK; ^7^ University of Southampton, UK

**Keywords:** Body composition, Frailty, Laparotomy, Emergency, General surgery, Mortality

## Abstract

**Introduction:**

Emergency surgical admissions represent the most unwell patients admitted to any hospital. Frailty and body composition independently identify risk of adverse outcomes but are seldom combined to predict outcomes in emergency patients. We aim to determine the relationships between frailty, body composition analyses (BCA) and mortality in an undifferentiated emergency general surgical patient population.

**Method:**

A prospective, multicentre observational cohort study of patients admitted with emergency surgical pathology was conducted in eight hospitals. BCA were performed at L3 vertebrae using computed tomography images to quantify sarcopenia and myosteatosis. Sex-specific BCA cut-off values were determined by our previous study. Reported Edmonton Frail Scale (REFS) values ≥8 identified frailty. The primary outcomes were all-cause 30-day and 1-year mortality. Multivariable logistic regression was utilised to explore predictive relationships between frailty, BCA, mortality and independent discharge.

**Results:**

A total of 194 patients were included; 24% were frail, 25% were sarcopenic and 23% myosteatotic. Some 61% of patients underwent an emergency laparotomy. Frail patients were more likely to be sarcopenic (20.4% vs 40.4%; *p* = 0.011) and myosteatotic (27.2% vs 51.1%; *p* = 0.004). Thirty-day and 1-year mortality was 5.2% and 15.5%, respectively; 30-day mortality was two times higher in the frail group (4.1% vs 8.5%; *p* = 0.414), and three times higher at 1 year (10.2% vs 31.9%; *p* = 0.001). Age (odds ratio [OR] 1.06; *p* = 0.001), sarcopenia (OR 2.88; *p* = 0.047) and frailty (OR 4.13; *p* = 0.001) were associated with 1-year mortality. Only 55.3% of frail patients were discharged home independently compared with 88.4% non-frail patients (*p* < 0.001). One-year mortality was greater in those with frailty and/or BCA abnormalities than in those without (28.8% vs 9.6%; *p* = 0.003).

**Conclusion:**

Frailty, sarcopenia and myosteatosis contribute significantly to adverse outcomes.

## Introduction

As western populations age, the prevalence of frailty is increasing. Ever improving surgical, radiological and anaesthetic techniques allow treatment access to more elderly, comorbid and frail patients presenting with emergency general surgical pathology.^1–4^ Some undergo emergency laparotomy, but others are treated conservatively. Frailty is a syndrome of decreased physiological reserve and decreased resilience to stressors resulting from a cumulative decline across a number of body systems highly prevalent in this patient group.^5–7^ Although definitions of frailty refer to older patients, younger adults can fulfil the criteria for frailty.^8^ Frail individuals undergoing surgery or suffering illness are at an increased risk of adverse outcomes than non-frail age-matched contemporaries.^3,4^

Unplanned or emergency admissions to hospital make up around 50% of general surgical workloads.^9^ These patients have historically been among the highest risk cohorts for morbidity and mortality, and the subject of much effort to improve outcomes.^10,11^ A key method of improving care has been the adoption of evidence-based care pathways promoting preoperative risk stratification, consultant-delivered surgery and critical care admission. In the United Kingdom (UK), the National Emergency Laparotomy Audit (NELA) risk calculator provides predicted morbidity and mortality risk that assists in shared decision making between clinicians and patients, and allows pre-emptive resource allocation for those at highest risk of poor postoperative outcomes.^10^ Commonly used prediction models are based on physiological, demographic and disease parameters, and do not include objective measures of frailty as part of their algorithms. More than half of all patients undergoing emergency laparotomy in the UK were aged 65 and older; however, fewer than one-third had early geriatric-led preoperative input.^10^ Patients undergoing similar emergency surgical pathology who have been treated conservatively are not captured in NELA, and consequently have not been the focus of research or quality improvement.^11,12^

Frailty may be assessed using a range of validated scoring systems including subjective and objective markers, though with substantial score disagreement and no single tool shown to be superior to another.^3,13,14^ Frailty scoring incorporated in the UK primary care systems and the US National Surgical Quality Improvement Program database shows strong associations between frailty and postoperative mortality; however, these are dominated by comorbidity variables.^15,16^ Some require the completion of physical tasks, such as timed get-up-and-go or a grip strength test, which are unsuitable for the acutely unwell patient. In both clinical care and research settings, frailty assessments are challenging in acute situations that typically fall out-of-hours with the need for urgent care and prompt decisions.

Body composition analyses (BCA) are an alternative and are potentially complementary to frailty assessments.^17^ BCA have the advantage of being opportunistically calculable from standard-of-care computed tomography (CT) scan images identifying poor quality (myosteatosis) or quantity (sarcopenia) of skeletal muscle tissue. Sarcopenia, a progressive and generalised skeletal muscle disorder, has been shown to be one of the best independent predictors of frailty in elective settings and offers the potential to be combined into patient assessments.^3,18–20^ Importantly, there remains a paucity of evidence relating to the emergency surgical setting and, to our knowledge, no data on their relationships and coexistence.

Frailty studies such as the UK Emergency Laparotomy and Frailty Study (ELF) recruited only patients aged over 65 undergoing emergency laparotomy.^21^ Although the prevalence of frailty increases with age, elderly people are not by definition frail, and conversely, young people may exhibit frailty. Multiple studies have shown that age-related cut-offs are not suitable because frailty and sarcopenia are present in patients of all ages.^22–24^ In a similar manner, although a frailer patient is more likely to have a larger number of comorbidities, frailty can be seen in the absence of comorbidities.^6^ Therefore, this study aimed to prospectively evaluate validated measures of frailty and BCA together, in an undifferentiated emergency general surgical patient cohort, with 1-year follow-up for mortality, to improve adverse outcome risk prediction (mortality and discharge destination) and inform preoperative shared decision making.

## Methods

This prospective, multicentre observational cohort study was conducted in eight UK National Health Service hospitals, received ethical approval from the UK Health Research Authority (18/NI/0094) and was registered with clinicaltrials.gov (NCT03534765).

### Inclusion criteria

All adult patients admitted under the care of an emergency general surgical team were screened and potentially eligible if they underwent a CT scan including the third lumbar vertebrae (L3) level, performed as part of their standard care, and diagnosis of an underlying pathology meeting criteria for inclusion in the UK NELA database. Briefly, all emergency admissions for gastrointestinal pathology are included except for appendicitis, uncomplicated hernia, biliary, vascular or gynaecological pathology (www.nela.org.uk/criteria). Patients provided written informed consent, but where their emergency care needs prevented timely approach or sufficient time to consider participation, inclusion remained possible if the clinician and research teams together with the patient and their friends, families and caregivers saw no objection to observational, anonymised data collection, with post-hoc consent. Patients were included regardless of treatment delivered; however, those receiving palliative and end-of-life care were excluded from analyses.

### Data collection

Data were prospectively recorded and stored using the secure REDCap online database system. Data points collected included age, sex, comorbidity data to allow calculation of the Charlson Comorbidity Index (CCI), CT findings, decision on management (surgical or conservative) and nonsurgical interventions administered.

On admission, a Reported Edmonton Frail Scale (REFS)^25^ assessment was performed and used to dichotomise patients into non-frail (REFS <8) and frail (REFS ≥8) groupings. REFS is designed for use in those who are currently unwell and may not be at their baseline function. It incorporates ten domains of frailty including cognition, balance and mobility to give a score from 0 to 17. REFS has been previously validated and shown to have excellent inter-assessor reliability, and does not require participants to complete a physical task.^5,7,25–27^ As such, REFS is a widely used, easy to complete, bedside tool suitable for use in acute settings. Frailty results were available to the responsible clinical teams, but they were blind to BCA results.

For patients undergoing an emergency laparotomy, the comprehensive data set submitted as part of the UK NELA audit database was captured. After discharge patients were followed for 1 year. Mortality was centrally assessed at 30 days and 1 year using NHS Digital Summary Care records, which link with national primary care mortality data. Length of hospital stay, unplanned readmissions, discharge destination and patient location at 1 year was also prospectively collected. Discharge destination was checked with a telephone follow-up call to relatives, carers or the patient at year 1.

### Body composition analysis

Image analyses were carried out as per our previous reports.^28^ Briefly, minimum CT image parameters at each site were ensured (5mm slice thickness, 120kVP and ∼290mA).^29^ Analyses were performed by a trained individual blinded to all patient outcome data. A single anonymised axial slice at the L3 level was selected for each patient. SliceOmatic v5.0 software (Tomovision, Magog, Canada) for Microsoft Windows® was utilised with predefined Hounsfield Unit (HU) ranges: −29 to 150HU for skeletal muscle (SM), −190 to −30HU for subcutaneous and intramuscular adipose tissue (SAT and IMAT), and −150 to −50HU for visceral adipose tissue (VAT).^28^ Cross-sectional areas (cm2) were corrected for patient height squared, to calculate the L3 index (in cm^2^/m^2^) for the skeletal muscle (SMI), subcutaneous (SATI) and visceral adipose tissue (VATI). Mean radiation attenuation (RA) was also assessed for all tissues in HU.

### Data analysis

To differentiate between low vs normal and high values for the body composition data, cut-off values representing the lower tertile, as defined in our recent study in a similar population, were used. SMI values <38.9 in males and <33.7 in females were defined as sarcopenia, with SM-RA values <29.3HU for males and <24.2HU for females defined as myosteatosis, as in our previous study.^28^ Patient groups were compared using chi-squared, Mann–Whitney *U* and Kruskal–Wallis testing. Results are presented as absolute numbers with percentages and as medians with interquartile ranges (IQR) unless stated otherwise. The primary outcomes of this study were all-cause mortality at 30 days and 1 year, and the relationship and coexistence of frailty and abnormal BCA. Discharge destination was a secondary outcome. A multivariable logistic regression model was utilised to explore factors predictive for 30-day and 1-year mortality and being discharged home.

## Results

**Figure 1:**
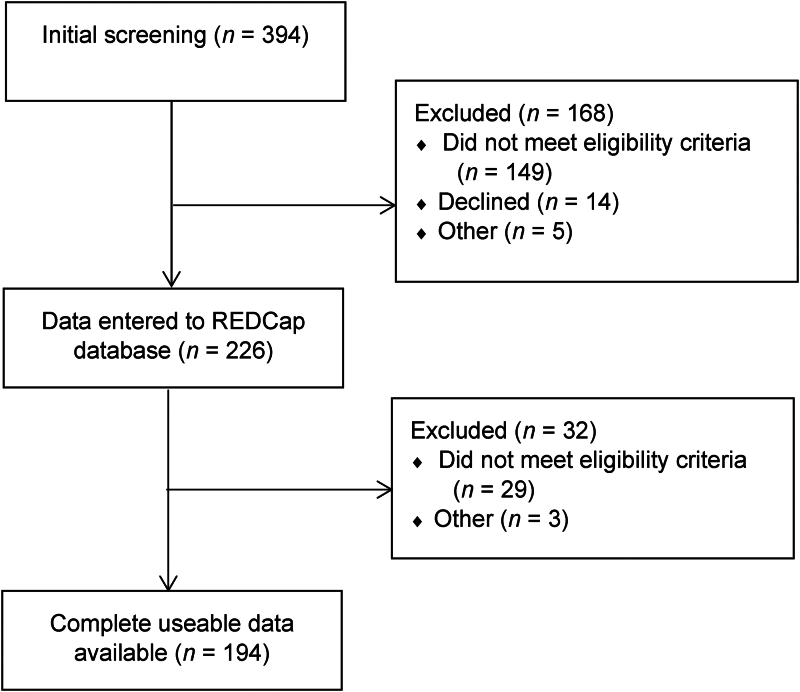
Study participant flow chart

A total of 394 patients were screened and 226 were consented into the study. A minority were subsequentially found to be ineligible or the CT images were of insufficient quality for analysis. Ultimately, 194 patients were included in the study (Figure 1). Of these, 46.4% were male and the median age was 70 (57.3–80.3) years with 25.3% being aged 80 years or older. More than half underwent surgical intervention (60.8%) with the remainder treated conservatively.

**Figure 2:**
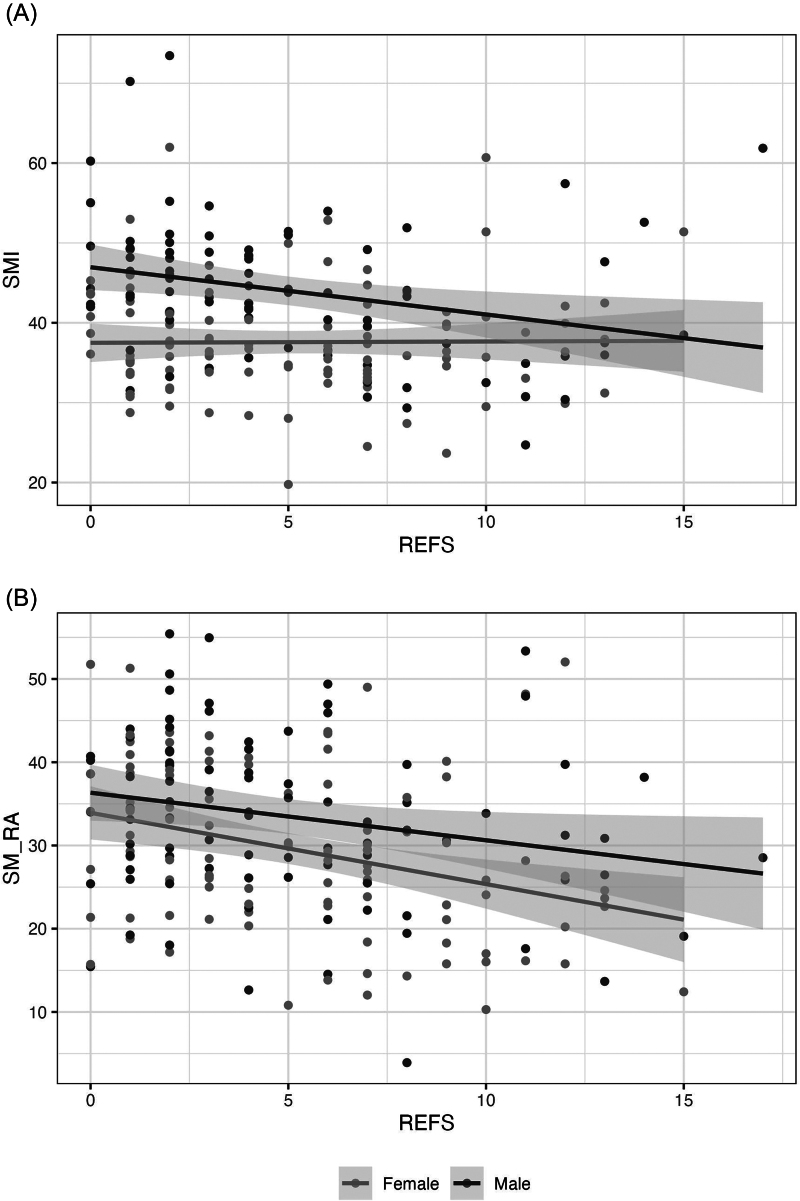
Scatterplots displaying numerical Reported Edmonton Frail Scale (REFS) results and sarcopenia (SMI) (A) and myosteatosis (SM-RA) (B) with linear regression lines for each sex with 95% confidence intervals. Except for females and sarcopenia, weak negative correlation was observed between decreasing body composition data and increasing REFS frailty scores (SMI males −0.13 [−0.23 to −0.03, *p* = 0.010]; SMI females (0.00 [−0.10 to 0.11, *p* = 0.930]; SM-RA males −0.09 [−0.17 to −0.01, *p* = 0.035]; SM-RA females −0.13 [−0.20 to −0.05, *p* = 0.001)).

Based on REFS results there were 147 (75.8%, REFS <8) non-frail and 47 (24.2%) frail patients. Full patient data dichotomised by frailty are shown in Table 1. Frail patients were older (68.0 [56.5–76.5] vs 77.0 [61.5–84.5] years, *p* = 0.004). There were no differences in the diagnoses between frail and non-frail groups or the observed rates of surgical intervention (60.5% vs 61.7%, *p* = 1). BCA showed 25.3% of the cohort were sarcopenic and 22.7% had myosteatosis. Full BCA data are presented in supplementary Tables 1 and 2. Frail patients were significantly more likely to be sarcopenic (20.4% vs 40.4%, *p* = 0.011) and have myosteatosis (27.2% vs 51.1%, *p* = 0.004). However, 59.6% of frail patients were not sarcopenic and 48.9% were not shown to have myosteatosis. Significant linear relationships between decreasing SM-RA and increasing frailty scores was identified (SM-RA males −0.09 [−0.17 to −0.01, *p* = 0.035], SM-RA females −0.13 [−0.20 to −0.05, *p* = 0.001]), but no clinically or statistically significant association between sarcopenia and frailty scores was observed (SMI males −0.13 [−0.23 to −0.03, *p* = 0.010], SMI females (0.00 [−0.10 to 0.11, *p* = 0.930[ (Figure 2).

**Table 1:** Demographic and diagnostic data for frail and non-frail groups defined by Reported Edmonton Frail Scale (REFS) result generated at time of acute surgical admission

REFS-defined frailty	Non-frail	Frail	*p*-value
Total *n* (%)	147 (75.8)	47 (24.2)	
Age*	68.0 (56.5–76.5)	77.0 (61.5–84.5)	**0.004**
Sex			
Female	77 (52.4)	27 (57.4)	0.661
Male	70 (47.6)	20 (42.6)	
Body mass index*	25.8 (22.6–28.8)	24.3 (20.9–29.5)	0.177
ASA*	2.0 (2.0–3.0)	3.0 (3.0–4.0)	**0.001**
Sarcopenia			
No	117 (79.6)	28 (59.6)	**0.011**
Yes	30 (20.4)	19 (40.4)	
Myosteatosis			
No	107 (72.8)	23 (48.9)	**0.004**
Yes	40 (27.2)	24 (51.1)	
Diagnosis			
Abscess	3 (2.0)	3 (6.4)	0.636
Adhesions	30 (20.4)	8 (17.0)	
Postoperative complication	3 (2.0)	1 (2.1)	
Colitis	10 (6.8)	2 (4.3)	
Diverticulitis	16 (10.9)	1 (2.1)	
Incarcerated hernia	15 (10.2)	6 (12.8)	
Intestinal ischaemia	5 (3.4)	1 (2.1)	
Intra-abdominal malignancy	19 (12.9)	7 (14.9)	
Intestinal perforation	25 (17.0)	9 (19.1)	
Other	21 (14.3)	9 (19.1)	
Treatment			
Surgery	89 (60.5)	29 (61.7)	1
Nonoperative	58 (39.5)	18 (38.3)	

Patients were dichotomised into non-frail (REFS <8) and frail (REFS ≥8) groups. Skeletal muscle mass index (SMI) <38.9 in males and SMI <33.7 in females defined as sarcopenia. Skeletal muscle radiation attenuation (SM-RA) <29.3 HU for males and SM-RA <24.2 Hounsfield units for females defined as myosteatosis. ASA = American Society of Anesthesiologists physical status classification system. Values are given as *n* (%), except *median (interquartile range).

**Table 2:** Patient outcomes after emergency surgical care displayed by Reported Edmonton Frail Scale (REFS) defined frailty

REFS-defined frailty	Non-frail	Frail	*p*-value
Total *n* (%)	147 (75.8)	47 (24.2)	
Unplanned ICU admission			
No	125 (98.4)	36 (90.0)	**0.044**
Yes	2 (1.6)	4 (10.0)	
Length of stay (days)*	8.0 (5–12)	12.5 (5–20.5)	**0.019**
Discharge destination			
Home: independent	130 (88.4)	26 (55.3)	**<0.001**
Home: carers	6 (4.1)	6 (12.8)	
Nursing home	2 (1.4)	2 (4.3)	
Hospice	1 (0.7)	0 (0.0)	
Other hospital	4 (2.7)	8 (17.0)	
Unknown	1 (0.7)	1 (2.1)	
Died in hospital	3 (2.0)	4 (8.5)	
Readmission			
No	119 (81.0)	35 (74.5)	0.454
Yes	28 (19.0)	12 (25.5)	
30-Day mortality			
No	141 (95.9)	43 (91.5)	0.414
Yes	6 (4.1)	4 (8.5)	
1-Year mortality			
No	132 (89.8)	32 (68.1)	**0.001**
Yes	15 (10.2)	15 (31.9)	

ICU = intensive care unit. Values are given as *n* (%), except *median (interquartile range).

Overall, 30-day and 1-year mortality was 5.2% and 15.5%, respectively. Higher in-hospital mortality was observed in the frail cohort (2% vs 8.5%, *p* < 0.001). There was a clinical but not statistical difference in 30-day mortality between non-frail and frail groups (4.1% vs 8.5%; *p* = 0.414). However, at 1 year nearly one-third of frail patients had died (10.2% vs 31.9%; *p* = 0.001) (Table 2). Univariable regression analysis showed only age (odds ratio [OR] 1.07, 95% confidence interval [CI] 1.02–1.15; *p* = 0.020) was independently related to 30-day mortality (Table 3). On assessment of variables related to 1-year mortality, age (OR 1.06, 95% CI 1.03–1.1; *p* = 0.001), sarcopenia (OR 2.88, 95% CI 0.99–5.15; *p* = 0.047) and frailty (OR 4.13, 95% CI 1.83–9.39; *p* = 0.001) were associated with not surviving. Only age (OR 1.06, 95% CI 1.06–1.11; *p* = 0.009) was related to 1-year mortality on multivariable regression analyses (Table 4).

**Table 3:** Univariate and multivariate analysis for 30-day mortality risk factors

30-Day mortality	No	Yes	OR (univariable)	OR (multivariable)
Age*	67.7 (14.3)	79.1 (11.3)	1.07 (1.02–1.15, *p* = **0.020**)	1.06 (0.98–1.16, *p* = 0.162)
Sex				
Female	98 (94.2)	6 (5.8)	–	–
Male	86 (95.6)	4 (4.4)	0.76 (0.19–2.75, *p* = 0.678)	1.28 (0.20–8.24, *p* = 0.788)
BMI*	26.5 (6.2)	24.9 (5.8)	0.95 (0.82–1.06, *p* = 0.458)	0.92 (0.71–1.09, *p* = 0.438)
ASA*	3.4 (2.5)	4.2 (1.8)	1.12 (0.88–1.38, *p* = 0.309)	0.98 (0.58–1.60, *p* = 0.917)
Sarcopenia				
No	140 (96.6)	5 (3.4)	–	–
Yes	44 (89.8)	5 (10.2)	3.18 (0.85–11.93, *p* = 0.078)	2.09 (0.28–14.43, *p* = 0.445)
Myosteatosis				
No	124 (95.4)	6 (4.6)	–	–
Yes	60 (93.8)	4 (6.2)	1.38 (0.34–5.00, *p* = 0.630)	0.57 (0.06–4.07, *p* = 0.590)
Frail				
No	141 (95.9)	6 (4.1)	–	–
Yes	43 (91.5)	4 (8.5)	2.19 (0.54–8.01, *p* = 0.242)	0.40 (0.02–2.86, *p* = 0.426)
Treatment				
Surgery	113 (95.8)	5 (4.2)	–	–
Nonoperative	71 (93.4)	5 (6.6)	1.59 (0.43–5.91, *p* = 0.475)	1.62 (0.10–12.89, *p* = 0.674)

ASA = American Society of Anesthesiologists physical status classification system; BMI = body mass index; OR = odds ratio. Values are given as *n* (%), except *mean (SD).

**Table 4:** Univariate and multivariate analysis for 1-year mortality risk factors

1-Year mortality	No	Yes	OR (univariable)	OR (multivariable)
Age*	66.7 (14.1)	76.7 (13.1)	1.06 (1.03–1.10, *p* **= 0.001**)	1.06 (1.02–1.11, *p* **= 0.009**)
Sex				
Female	89 (85.6)	15 (14.4)	–	–
Male	75 (83.3)	15 (16.7)	1.19 (0.54–2.60, *p* = 0.667)	1.82 (0.67–5.14, *p* = 0.247)
BMI*	26.7 (6.3)	24.7 (5.3)	0.94 (0.86–1.01, *p* = 0.120)	0.97 (0.87–1.06, *p* = 0.576)
ASA*	3.3 (2.5)	3.9 (2.2)	1.09 (0.94–1.26, *p* = 0.214)	1.09 (0.82–1.44, *p* = 0.533)
Sarcopenia				
No	127 (87.6)	18 (12.4)	–	–
Yes	37 (75.5)	12 (24.5)	2.88 (0.99–5.15, *p* **= 0.047**)	1.44 (0.49–4.06, *p* = 0.492)
Myosteatosis				
No	112 (86.2)	18 (13.8)	–	–
Yes	52 (81.2)	12 (18.8)	1.44 (0.63–3.18, *p* = 0.376)	0.90 (0.29–2.65, *p* = 0.849)
Frail				
No	132 (89.8)	15 (10.2)	–	–
Yes	32 (68.1)	15 (31.9)	4.13 (1.83–9.39, *p* **= 0.001**)	2.62 (0.93–7.31, *p* = 0.065)
Treatment				
Surgery	101 (85.6)	17 (14.4)	–	–
Nonoperative	63 (82.9)	13 (17.1)	1.23 (0.55–2.69, *p* = 0.612)	1.03 (0.26–3.54, *p* = 0.964)

ASA = American Society of Anesthesiologists physical status classification system; BMI = body mass index; OR = odds ratio. Values are given as *n* (%), except *mean (SD).

Frailty was also shown to have significant impact on other aspects of patients’ outcomes. Length of stay increased compared with non-frail counterparts (8 days [5–12] vs 12.5 [5–20.5], *p* = 0.019) and frail patients were more likely to have an unplanned admission to intensive care (1.6% vs 10.0%, *p* = 0.044). The strongest association identified was discharge destination, with only 55.3% of frail patients discharged home independently compared with 88.4% of non-frail patients (*p* < 0.001). Frail patients showed a higher discharge rate to other hospitals, to nursing care facilities or to their own home but with enhanced care needs than non-frail individuals (OR 5.84 [2.19–16.25], *p* < 0.001) (Table 5).

**Table 5:** Univariate and multivariate analysis for discharge destination of home without additional care support

Discharge destination: home	Yes	No	OR (univariable)	OR (multivariable)
Age*	66.9 (14.3)	76.3 (12.3)	1.06 (1.02–1.10, *p* **= 0.009**)	1.05 (1.00–1.12, *p* = 0.081)
Sex				
Female	88 (88.0)	12 (12.0)	–	–
Male	80 (92.0)	7 (8.0)	0.64 (0.23–1.68, *p* = 0.375)	1.13 (0.30–4.24, *p* = 0.858)
BMI*	26.3 (5.6)	27.3 (9.8)	1.02 (0.95–1.09, *p* = 0.522)	1.12 (1.02–1.23, *p* **= 0.019**)
ASA*	3.4 (2.5)	3.5 (2.1)	1.03 (0.84–1.22, *p* = 0.778)	1.54 (0.96–2.95, *p* = 0.112)
Sarcopenia				
No	128 (90.1)	14 (9.9)	–	–
Yes	40 (88.9)	5 (11.1)	1.14 (0.35–3.20, *p* = 0.809)	0.72 (0.16–2.81, *p* = 0.646)
Myosteatosis				
No	112 (88.2)	15 (11.8)	–	–
Yes	56 (93.3)	4 (6.7)	0.53 (0.15–1.55, *p* = 0.283)	0.10 (0.02–0.45, *p* **= 0.006**)
Frail				
No	136 (94.4)	8 (5.6)	–	–
Yes	32 (74.4)	11 (25.6)	5.84 (2.19–16.25, *p* **< 0.001**)	11.06 (2.76–53.40, *p* **= 0.001**)
Treatment				
Surgery	98 (86.0)	16 (14.0)	–	–
Nonoperative	70 (95.9)	3 (4.1)	0.26 (0.06–0.83, *p* **= 0.039**)	0.03 (0.00–0.34, *p* **= 0.027**)

ASA = American Society of Anesthesiologists physical status classification system; BMI = body mass index; OR = odds ratio. Values are given as *n* (%), except *mean (SD).

**Figure 3:**
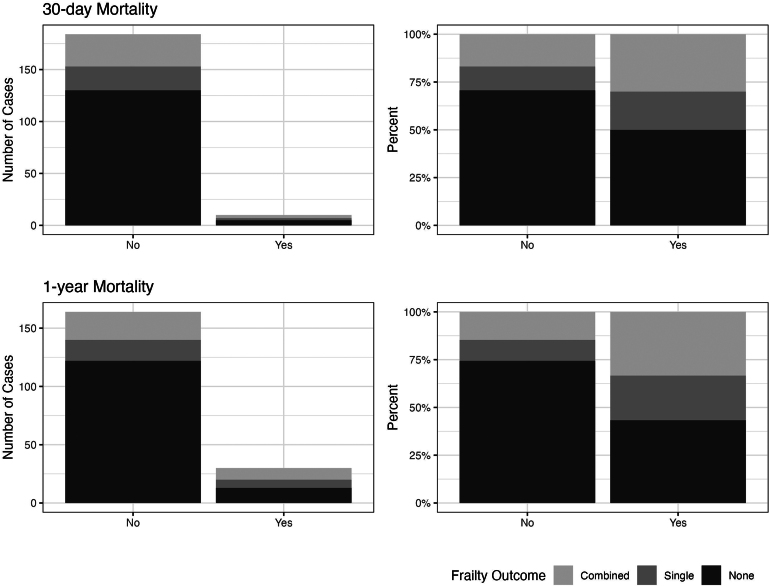
Graphical depiction of mortality data for both 30-day and 1-year mortality displayed by numbers and percentage and the presence of both frailty and abnormal body composition analyses (light), frailty or abnormal body composition analyses (BCA) results (medium) and neither risk factor (dark). Frail and abnormal BCA patients were disproportionately observed to have died at one year (*p* = 0.003).

Frailty and body composition data were combined and compared with 30-day and 1-year mortality (Table 6, Figure 3). Although a low number of 30-day deaths were observed (*n* = 10, 5.2%), patients with the coexistence of frailty and abnormal BCA were clinically, but not statistically significantly, more likely to have died. One-year mortality was, however, greater in those with frailty and/or BCA abnormalities than in those without (28.8% vs 9.6%, *p* = 0.003) (Table 7, Figure 3).

**Table 6:** 30-Day mortality stratified by the presence of frailty and/or abnormal body composition analysis results or neither

30-Day mortality	Alive, *n* (%)	Died, *n* (%)	Total	*p*-value
Frail + Sarcopenia + Myosteatosis	31 (16.8)	3 (30)	34	0.381
Frail or Sarcopenia + Myosteatosis	23 (12.5)	2 (20)	25	
Neither	130 (70.7)	5 (50)	135	
Total	184 (94.8)	10 (5.2)	194	

**Table 7:** One-year mortality stratified by the presence of frailty and/or abnormal body composition analysis results or neither

1-Year mortality	Alive, *n* (%)	Died, *n* (%)	Total	*p*-value
Frail + Sarcopenia + Myosteatosis	24 (14.6)	10 (33.3)	34	**0.003**
Frail or Sarcopenia + Myosteatosis	18 (11)	7 (23.3)	25	
Neither	122 (74.4)	13 (43.3)	135	
Total	164 (84.5)	30 (15.5)	194	

## Discussion

We prospectively assessed both frailty and body composition using validated instruments in an unselected multicentre emergency general surgery cohort. This study was specifically designed to include patients of all ages and not just those undergoing surgery. This was to maximise generalisability and explore these clinically important groups, which are challenging to capture in prospective research and typically overlooked in the few available literature reports that understandably focus on elderly patients when studying frailty.^21^

The large-scale NELA initiative was commissioned in the UK aiming to enhance care for patients undergoing emergency laparotomy. Substantial quality improvements have been shown; however, emergency laparotomy remains a high-risk intervention with the eighth national report showing 9.2% in-hospital mortality, down from 11% in 2015. Only 31.8% of patients who were identified as being frail had perioperative geriatrician input.^10^ Twenty-four per cent of all admitted patients admitted to our acute surgery services were frail. Although this figure is in keeping with reports from comparable populations, our data set is significantly enriched by body composition, reliable discharge destination data follow-up and centrally verified 1-year mortality. Patients who were frail were more likely to be sarcopenic, but surprisingly we observed around half of frail patients were not sarcopenic and so interdependence should not be assumed. Despite this, linear relationships were seen between declining body composition data and increasing REFS scores.

As expected, frailty and/or sarcopenia were associated with a range of poor outcomes in this study, with age, sarcopenia and frailty independently associated with 1-year mortality. Routine measurement of frailty and body composition at time of acute admission could prove informative to patients and clinicians at no additional cost because automatic assessments of body composition will become embedded within radiology software systems. As well as predicting higher risks and hospital resource usage, we saw frailty associated with longer hospital stays and, critically, only half of frail patients being discharged home independently. This stark finding may assist the often-challenging shared decision-making processes when high-risk emergency interventions are being considered. In-hospital metrics such as morbidity, length of stay and short-term mortality are well understood and easier to record, but functional and post-discharge outcomes are of higher importance to patients and caregivers.^30^ As the populations of developed nations age, the need for reliable and reproducible risk measures becomes ever greater and our approach may represent a route towards this goal. We observed frailty and sarcopenia across the cohort, and therefore both tools might represent valuable baseline assessments upon emergency presentation and inform perioperative decision making based on objective data at little extra cost or delay.

A review on the impact of frailty on surgical outcomes showed double the risk of major morbidity, three times the risk of mortality within 1 year, and six times the risk of mortality within 90 days.^3^ A large Canadian study showed that a 10% increase in the preoperative Frailty Index increased the adjusted odds of mortality by 2.3 after emergency surgery, with high rates of institutional discharge.^31^ Recently the ELF Study Group found that Clinical Frailty Scores were associated with risk of postoperative morbidity and mortality and were independent of age.^21^ The importance of individualised management based on a patient’s degree of frailty and comorbidity irrespective of age is paramount.

Although both frailty and sarcopenia in the emergency setting are non-modifiable risk factors, we are unaware of any prior study that has simultaneously explored both. Their addition to traditional risk stratification metrics such as age, physiological disturbance and serological markers might provide a gateway to personalised emergency patient care or the consideration of nonoperative management. A window of opportunity frequently exists to explore shared decision making, provide appropriate expectation management to patients and relatives and provide counsel regarding meaningful patient outcomes. A recently reported systematic review identified 11 studies exploring sarcopenia and emergency surgery outcomes but notably all were retrospective, and all patients had undergone surgery.^32^ All were seen to have moderate or high bias risks, limiting the findings. We note not dissimilar ORs in our prospective cohort in which methodological care was taken to overcome these previously identified issues. A large UK prospective multicentre study also assessed all patients’ acute surgery admission, with 62% not undergoing surgery, and found frailty to exist in all age groups and be independently associated with age with a linear relationship with 90-day mortality.^33^ The proportion of patients diagnosed as frail in our study (24.2%) is similar to the ELF study (20%), which used clinical frailty scoring in patients aged over 65 years receiving emergency laparotomies.^21^ Our additional body composition data present new insights, show sarcopenia or frailty should not be assumed to be mutually present or absent, and their combination has synergistic advantages over singular analysis.

### Study limitations

Unfortunately, we were unable to replicate the strength of association found in our previous study,^28^ in which both sarcopenia and myosteatosis were strong predictors of 30-day and 1-year mortality. The main difference here is the inclusion of conservatively treated patients; however, here both frailty and sarcopenia utilising the same cut-off values were significantly associated with 1-year mortality. Capturing patients admitted via an emergency surgical route, often presenting out-of-hours, with urgent care needs, provided consenting, REFS completion and CT imaging challenges, leading to a relatively high screen failure rate. Despite these challenges, this surgical trainee-led and delivered study adopted robust screening and recruitment protocols, with high REFS completions rates, few exclusions of CTs because of poor images and efficient recruitment to time and target. A further limitation was the absence of postoperative complication data that were challenging to obtain reliably. Body composition analysis is a labour-intensive undertaking including the need for specialist software and training, which currently limits it to the research setting. Efforts are underway to automate the process, which may allow routine analysis in the future. Finally, we were unable to include those who received end-of-life care with palliative intent irrespective of their pathology and frailty and/or sarcopenic statuses. Although this would have been informative, seeking permission for consent and REFS completion in this context was considered inappropriate and unlikely to alter decision making.

## Conclusion

In summary, both frailty and body composition abnormalities are commonly encountered in unselected general surgical emergency patients, but are not interdependent. Because they represent risk factors for adverse outcomes, routine measurement and the clinical utility of both could present advantages to shared decision making and risk-counselling for patients and emergency surgical teams, especially when discussing 1-year survival, discharge destinations and independent living after major emergency surgery.

## Data Availability

The authors are happy to share raw body composition data and master database for the fully anonymised study data.
